# Pharmacokinetics and Pharmacodynamics of Darunavir and Etravirine in HIV-1–Infected, Treatment-Experienced Patients in the Gender, Race, and Clinical Experience (GRACE) Trial

**DOI:** 10.1155/2012/186987

**Published:** 2012-03-21

**Authors:** Thomas Kakuda, Vanitha Sekar, Peter Vis, Bruce Coate, Robert Ryan, David Anderson, Guy De La Rosa, Joseph Mrus

**Affiliations:** ^1^Janssen Research & Development, LLC., Clinical Pharmacology, 1125 Trenton-Harbourton Road, Titusville, NJ 08560, USA; ^2^Janssen Infectious Diseases BVBA, Clinical Pharmacology, Turnhoutseweg 30, B-2340 Beerse, Belgium; ^3^Janssen Research & Development, LLC., Medical Affairs Biometrics, 1125 Trenton-Harbourton Road, Titusville, NJ 08560, USA; ^4^Janssen Services, LLC., Clinical Affairs, 1125 Trenton-Harbourton Road, Titusville, NJ 08560, USA

## Abstract

*Objectives*. Evaluation of pharmacokinetics and pharmacodynamics of darunavir and etravirine among HIV-1–infected, treatment-experienced adults from GRACE, by sex and race. *Methods*. Patients received darunavir/ritonavir 600/100mg twice daily plus other antiretrovirals, which could include etravirine 200mg twice daily. Population pharmacokinetics for darunavir and etravirine were determined over 48 weeks and relationships assessed with virologic response and safety. Rich sampling for darunavir, etravirine, and ritonavir was collected in a substudy at weeks 4, 24, and 48. *Results*. Pharmacokinetics were estimated in 376 patients for darunavir and 190 patients for etravirine. Median darunavir AUC_12h_ and C_0h_ were 60,642ng*·*h/mL and 3624ng/mL, respectively; and for etravirine were 4183ng *·* h/mL and 280ng/mL, respectively. There were no differences in darunavir or etravirine AUC_12h_ or C_0h_ by sex or race. Age, body weight, or use of etravirine did not affect darunavir exposure. No relationships were seen between darunavir pharmacokinetics and efficacy or safety. Patients with etravirine exposure in the lowest quartile generally had lower response rates. Rich sampling showed no time-dependent relationship for darunavir, etravirine, or ritonavir exposure over 48 weeks. *Conclusions*. Population pharmacokinetics showed no relevant differences in darunavir or etravirine exposure by assessed covariates. Lower etravirine exposures were associated with lower response rates.

## 1. Introduction

Differences in antiretroviral pharmacokinetic parameters between women and men, caused by variables such as body weight, plasma volume, and cytochrome P450 activity, could lead to different drug concentrations and toxicity profiles between sexes [1–3]. Previous pharmacokinetic data from the antiretroviral therapy with TMC114 examined in naïve subjects (ARTEMIS) and TMC114/r in treatment-experienced patients naïve to lopinavir (TITAN) trials, which studied 343 treatment-naïve and 298 treatment-experienced patients receiving darunavir/ritonavir, respectively, have demonstrated small, nonclinically relevant differences in darunavir pharmacokinetic parameters between women and men and across races [4, 5]. The once-daily darunavir in treatment-experienced patients (ODIN) trial, which studied 294 patients receiving once-daily darunavir versus 296 patients receiving twice-daily darunavir, found that women had higher exposures than men, and Asian patients had lower exposure than white patients; however, these differences were not considered clinically significant [6]. Data from the pooled TMC125 to demonstrate undetectable viral load in patients experienced with antiretroviral therapy (DUET)-1 and DUET-2 trials, which compared treatment with etravirine (*n* = 599) versus placebo (*n* = 604) in treatment-experienced patients, did not demonstrate any sex or racial differences in etravirine pharmacokinetic parameters [7]. These trials, however, were not specifically designed to investigate sex-based or race-based differences in darunavir or etravirine pharmacokinetics.

The gender, race, and clinical experience (GRACE) study was specifically designed to assess sex-based and race-based differences in the pharmacokinetics, efficacy, and safety of darunavir/ritonavir-based therapy in treatment-experienced, HIV-1–infected patients by enrolling a high proportion of women and people of color [8]. This paper presents the darunavir, ritonavir, and etravirine pharmacokinetic data from GRACE by sex and race, and the relationship of darunavir and etravirine pharmacokinetics with efficacy and safety, collected over 48 weeks. The relationship between extrinsic and intrinsic covariates with darunavir pharmacokinetics is also investigated.

## 2. Materials and Methods

### 2.1. Study Design and Treatment

GRACE was a 48-week, open-label, Phase IIIb study conducted at 65 study sites across the United States, Canada, and Puerto Rico. Treatment-experienced adults with HIV-1 RNA ≥1000 copies/mL received darunavir 600 mg coadministered with ritonavir 100 mg twice daily with other antiretrovirals, which could include etravirine 200 mg twice daily. The choice of additional antiretrovirals was based on resistance testing (virco^®^TYPE HIV-1). During enrollment, the virco^®^TYPE HIV-1 resistance test used did not include etravirine, which resulted in some patients with reduced susceptibility to etravirine receiving the drug. Subsequently, at the time of data analysis, baseline samples were reanalyzed using an updated version of the virco^®^TYPE HIV-1 resistance test interpretation, which included etravirine. The data referenced in this paper are those obtained from the updated analysis. Women who were pregnant were excluded from the study. Other inclusion/exclusion criteria and study visits have been described previously [8]. Human experimentation guidelines of the United States Department of Health and Human Services and the Declaration of Helsinki were followed in the conduct of this clinical research; the research protocol was reviewed and approved by institutional review boards for all 65 study sites; written informed consent was provided by all participants prior to study initiation. Details of the study design were registered at clinicaltrials.gov (ID: NCT00381303).

### 2.2. Pharmacokinetic Analysis

Sparse sampling for the determination of darunavir and etravirine (if applicable) pharmacokinetic parameters was performed at Weeks 4, 8, 24, and 48. Two samples were taken at Weeks 4 and 24, one immediately before intake of medication and one at least an hour after intake of medication. At Weeks 8 and 48, the samples could be taken at any time after intake of medication. Pharmacokinetics were considered evaluable if the sample had measurable darunavir and ritonavir or etravirine (if applicable) concentrations, and if the time of last intake or administration was known. Previously developed population pharmacokinetic models [7, 9] were applied to the sparse sampling data to derive empirical Bayesian estimates of darunavir and etravirine area under the plasma concentration–time curve (AUC_12h_) and trough concentration (C_0h_).

In a subset of consenting patients from the pharmacokinetic substudy, intensive blood sampling for darunavir, ritonavir, and etravirine (if applicable) was conducted over 12 hours; samples were collected before dose and 1, 2, 3, 4, 6, 9, and 12 hours after dose at Weeks 4, 24, and 48. Ritonavir concentrations were determined to assess adherence to that medication. Patients were required to have fasted for 10 hours before arrival at the testing site. A standardized breakfast was served at the facility, and medications were administered within 30 minutes of the meal. In order to be included in the intensive pharmacokinetic sampling, patients had to volunteer and already participate at a study site that was involved in the intensive pharmacokinetic analysis.

Plasma concentrations of darunavir, ritonavir, and etravirine in the main study and substudy were determined using a previously validated liquid chromatography–tandem mass spectrometry method; the lower limit of quantification was 10.0 ng/mL, 5.0 ng/mL, and 2.0 ng/mL for darunavir, ritonavir, and etravirine, respectively [10].

Relationships between darunavir and etravirine pharmacokinetics (Bayesian estimated AUC_12h_ and C_0h_) and virologic efficacy at Week 48, measured by change in log_10_ viral load (VL) from baseline and the proportion of patients achieving a VL less than 50 copies/mL, were assessed using analysis of covariance models. The impact of extrinsic and intrinsic covariates (use of etravirine [relationship with darunavir pharmacokinetics only] and use of tenofovir disoproxil fumarate [TDF], age, sex, race, body weight, and hepatitis B coinfection status) on darunavir and etravirine pharmacokinetics was explored graphically, using descriptive statistics and by analysis of covariance. Tenofovir disoproxil fumarate was included in the covariate analysis due to previous evaluations suggesting a drug-drug interaction with etravirine [11]. Relationships between darunavir and etravirine pharmacokinetics and safety (rash-, cardiac-, gastrointestinal-, liver-, lipid-, glucose-, psychiatric-, and nervous system–associated adverse events), including laboratory assessments, were investigated and are presented using descriptive statistics. Week 48 pharmacokinetic data were used to evaluate all relationships with efficacy, covariates, and safety.

## 3. Results

### 3.1. Patient Populations and Baseline Characteristics

GRACE enrolled a total of 429 patients, of whom 66.9% were women, 61.5% were black, 22.4% were Hispanic, and 15.2% were white. In the intent–to–treat time–to–loss of virologic response analysis of the overall population, 53.4% of patients achieved virologic response (HIV-RNA <50 copies/mL) after 48 weeks; women had a lower response compared with men (50.9% [confidence interval (CI) range: 45.1%–56.7%] and 58.5% [50.3%–66.6%], resp.), and black patients had a lower response rate compared with Hispanic and white patients (48.5% [42.5%–54.5%], 61.5% [51.7%–71.2%], and 60.0% [48.1%–71.9%], resp.) [8, 12]. Patients who received etravirine had slightly higher response rates than did the overall population. In the intent–to–treat time–to–loss of virologic response analysis of the etravirine population, 59.4% of patients achieved virologic response; women had a slightly lower response rate compared with men (58.0% [49.1%–66.9%] and 61.4% [51.2%–71.5%], resp.), and black patients had a lower response rate compared with Hispanic or white patients (55.6% [47.2%–64.1%], 69.4% [54.4%–84.5%], and 61.8% [45.4%–78.1%], resp.) [12, 13].

Of the 429 patients in the overall GRACE population, evaluable pharmacokinetic data from sparse sampling were available for 376 patients (Table 1). Among these patients, 66% (*n* = 248) were women, 60% (*n* = 226) were black, 22% (*n* = 84) were Hispanic, 17% (*n* = 62) were white, and 1% (*n* = 4) were Asian or other. In total, 37 patients—including 25 women, 12 men, 25 black patients, 10 Hispanic patients, and 2 white patients—underwent intensive pharmacokinetic sampling.

Of the 207 patients who received etravirine in addition to darunavir (Table 1), evaluable pharmacokinetic data from sparse sampling were available for 190 patients. These patients included 108 (57%) women, 122 (64%) black, 33 (17%) Hispanic, 31 (16%) white, and 4 (2%) Asian or other patients. Of the patients who received etravirine, 16 underwent intensive pharmacokinetic sampling, including 8 women, 11 black, 4 Hispanic, and 1 white patient.

### 3.2. Pharmacokinetics

#### 3.2.1. Population Pharmacokinetic Analyses over 48 Weeks

Among the 429 patients enrolled in this trial, 222 did not receive etravirine and 207 received at least one dose of etravirine. Based on pharmacokinetic data available for 376 patients, including both recipients and nonrecipients of etravirine, the median (range) darunavir AUC_12h_ and C_0h_ were 60,642 (26,117–128,790) ng*·*h/mL and 3624 (931–9570) ng/mL, respectively. Based on pharmacokinetic data available for 187 patients who did not receive etravirine, the median (range) darunavir AUC_12h_ and C_0h_ were 58,933 (26,117–128,790) ng*·*h/mL and 3489 (1036–9570) ng/mL, respectively. Based on pharmacokinetic data available for 189 patients who received etravirine, the median (range) darunavir AUC_12h_ and C_0h_ were 62,626 (30,960–109,410) ng*·*h/mL and 3806 (931–7473) ng/mL, respectively. In those patients who received etravirine, the median (range) etravirine AUC_12h_ and C_0h_ were 4183 (212–27,960) ng*·*h/mL and 280 (4–2211) ng/mL, respectively. Analysis of darunavir and etravirine pharmacokinetics by sex and race showed no clinically relevant difference in AUC_12h_ or C_0h_  between sexes or across races. Based on univariate analysis, hepatitis B co-infection status, age, body weight, or use of etravirine or TDF did not affect darunavir AUC_12h_ or C_0h_ (Table 2).

Patients with TDF in their background regimen had lower median etravirine exposure (AUC_12h_, 3998 ng*·*h/mL; C_0h_, 258 ng/mL) compared with those without TDF (AUC_12h_, 5051 ng*·*h/mL; C_0h_, 329 ng/mL), and patients with hepatitis B co-infection demonstrated a trend toward higher median etravirine exposures (AUC_12h_, 5504 ng*·*h/mL;  C_0h_, 382 ng/mL) compared with those without co-infection (AUC_12h_, 4141 ng*·*h/mL; C_0h_, 278 ng/mL). We further analyzed several of these covariates using an analysis of covariance (Table 3). Only age and female sex were statistically correlated with higher darunavir exposure; older age was also correlated with higher etravirine exposure. However, none of these associations were considered clinically relevant as evidenced by univariate analysis.

#### 3.2.2. Intensive Pharmacokinetic Analyses Over 48 Weeks

Intensive pharmacokinetic sampling showed no time-dependent relationship for darunavir, ritonavir, or etravirine exposure over 48 weeks; darunavir and etravirine intensive pharmacokinetic results were generally similar to the population pharmacokinetic results (Table 4). Mean plasma concentration–time profiles for darunavir were higher in women than in men, with an AUC_12h_ approximately 18%, 33%, and 14% higher in women than in men at Weeks 4, 24, and 48, respectively. Ritonavir AUC_12h_ was approximately 44% higher in women at Weeks 4 and 24 and 8% lower in women than in men at Week 48. Mean plasma concentration–time profiles for both darunavir and ritonavir slightly differed when comparing black and Hispanic patients. For darunavir, higher concentrations were observed for black patients at Weeks 4 and 48 than for Hispanic patients. When the intensive pharmacokinetic data for etravirine were broken down by sex or race, the sample sizes were too small to draw any definitive conclusions (Table 4).

#### 3.2.3. Relationship between Pharmacokinetics (Sparse Sampling) and Efficacy

**Figure 1:**
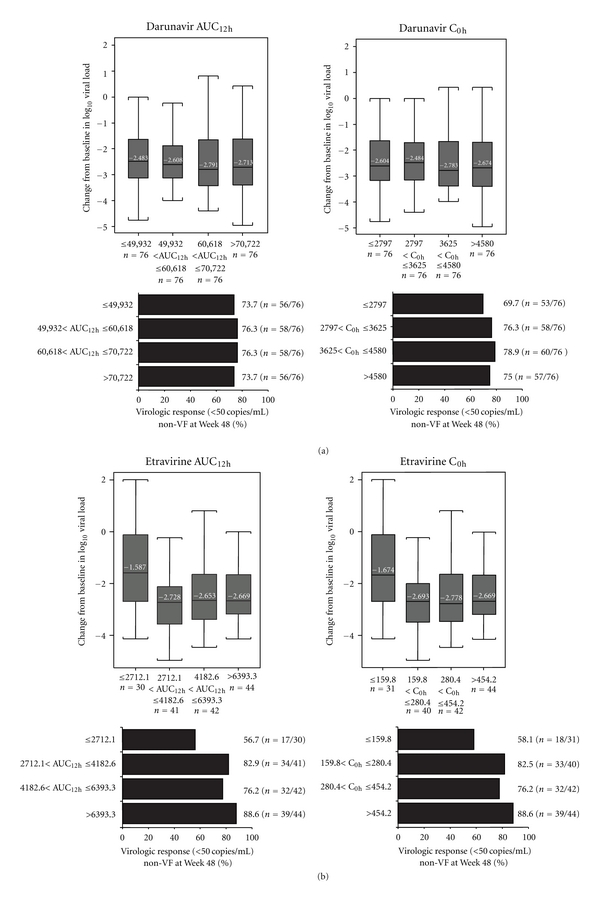
Change in log_10_ viral load from baseline to Week 48 (nonvirologic failure censored) and virologic response by quartile ranges of (a) darunavir AUC_12h_ and C_0h_ (sparse pharmacokinetic sampling; *n* = 376) and (b) etravirine AUC_12h_ and C_0h_ (sparse pharmacokinetic sampling;  *n* = 190). In Figures 1(a) and 1(b), the numbers within the boxplots represent the median values, the boxes represent the 25th and 75th percentiles, and the whiskers represent the highest and lowest value within 1.5 interquartile range. AUC_12h_: area under the plasma concentration–time curve over 12 hours; C_0h_: trough concentration; DRV: darunavir; non-VF: nonvirologic failure censored population; ETR: etravirine.

When the relationships between darunavir population pharmacokinetics and efficacy parameters were investigated, no relationships were observed between darunavir AUC_12h_ or C_0h_ values and the change in log_10_ VL from baseline to Week 48, or the proportion of patients achieving less than 50 copies/mL by Week 48 in the overall nonvirologic failure–censored population, which censored patients who discontinued for reasons other than virologic failure (Figure 1(a)). Furthermore, consistent with the above results, no relationship between darunavir Week 48 pharmacokinetics and change in VL or virologic response was seen by sex or race.

When the relationships between etravirine pharmacokinetics and efficacy parameters in the nonvirologic failure–censored population were investigated, patients with AUC_12h_ or C_0h_ in the lowest quartile at Week 48 had the smallest change in log_10_ VL from baseline to Week 48 (Figure 1(b)). These patients in the lowest quartile of AUC_12h_  or C_0h_ at Week 48 also demonstrated the lowest virologic response rates, compared with the other pharmacokinetic quartiles (Figure 1(b)).

#### 3.2.4. Relationship between Pharmacokinetics and Safety

When the relationships between darunavir and etravirine pharmacokinetic parameters and safety in the overall population were investigated, no apparent relationships were observed between darunavir or etravirine AUC_12h_ or C_0h_ and the incidence of rash-, cardiac-, gastrointestinal-, liver-, lipid-, glucose-, nervous system disorder–, or psychiatric disorder–associated adverse events (data not shown). Similarly, no relationships were seen between darunavir pharmacokinetics and safety parameters by sex or race (data not shown).

## 4. Discussion

Sex and race did not appear to substantially affect darunavir or etravirine exposure. Similar darunavir exposures have been observed in treatment-experienced patients from the performance of TMC114/r when evaluated in treatment- experienced patients with PI resistance (POWER 1, 2, and 3 and TITAN trials [4, 14]). Likewise, previous studies of etravirine pharmacokinetics have yielded median values similar to those seen here [7]. Furthermore, the ranges of darunavir and etravirine exposure observed in this study were numerically similar to those from previous studies [4, 7].

Although this study was not specifically powered to compare the effects of covariates on pharmacokinetics, all groups (e.g., women versus men) were well represented, allowing for meaningful comparisons. This study demonstrated that the pharmacokinetic exposure (i.e., AUC_12h_ and C_0h_) to darunavir was not substantially influenced by sex, race, age, body weight, hepatitis B co-infection status, or use of etravirine or TDF, similar to results from other studies of darunavir/ritonavir in treatment-experienced, HIV-infected patients [4, 5, 7, 10, 15]. Trials of other HIV protease inhibitors (PIs) have demonstrated an influence of various covariates on pharmacokinetic parameters. For instance, 2 studies have demonstrated significant differences in exposure of saquinavir and indinavir between women and men [16, 17]. In contrast to the darunavir pharmacokinetic results, patients using TDF or with hepatitis B co-infection demonstrated trends toward lower and higher etravirine exposure, respectively. Similar results were obtained in the DUET trials and were not deemed clinically relevant [7]. The effect of TDF use was as expected, based on a known drug-drug interaction [11], and it should be noted that use of TDF did not affect clinical outcomes in this trial [8].

Although no clinically relevant sex-based differences in population pharmacokinetic parameters were seen during this trial, an analysis of covariance (which showed statistical differences and intensive pharmacokinetic sampling in a subset of patients), did suggest a trend toward higher darunavir and ritonavir exposure in women compared with men. Similar results were seen in TITAN, which suggested that women had slightly higher darunavir exposures than men (~15% higher) and that black patients had slightly higher darunavir exposures than white patients (~8% higher); these differences were not considered clinically relevant. The trend toward increased darunavir exposure in women, observed in this trial and in TITAN, may be due to several factors, including, but not limited to, physiologic differences in protein binding, gastric motility, sex hormones, and/or *α*_1_-acid glycoprotein (AAG) levels [18]. Elevated AAG levels have been linked to increased PI binding and, therefore, exposure [19, 20]. Indeed, at baseline, women in the pharmacokinetic substudy of the GRACE trial had AAG levels approximately 12% higher than those of men [21]. Recently, a post hoc analysis of the GRACE pharmacokinetic Week 4 substudy investigated the relationship of plasma estrone sulfate (E3S), a sex hormone, with darunavir and ritonavir pharmacokinetics [22]. In this case, no differences were seen in the plasma concentrations of E3S between women and men in the substudy. Additionally, although E3S and darunavir were both substrates for the hepatic uptake transporter SLCO1B1, no relationship was seen between plasma concentrations of E3S and the pharmacokinetics of darunavir or ritonavir. In the current study, it is possible that unidentified differences in baseline physiology between the populations undergoing sparse (*n* = 376) or intensive (*n* = 37) pharmacokinetic sampling may also account for the fact that no sex-based difference was seen in the former population, in contrast to the small differences seen in the latter population. Although the sample sizes were too small to draw any definitive conclusions for the etravirine intensive pharmacokinetic data by sex or race, results did seem consistent with the population pharmacokinetic results of this and other trials [7].

Intensive pharmacokinetic sampling showed no time-dependent relationship for darunavir, ritonavir, or etravirine. As observed in other studies of darunavir/ritonavir in treatment-naïve and treatment-experienced patients [4, 14, 23], no relevant relationships between darunavir pharmacokinetic parameters and the safety or efficacy of darunavir/ritonavir-based therapy were observed at Week 48 in the overall population, by sex or by race. Week 48 was chosen for these comparisons because we wanted to investigate the correlation between steady-state drug exposure and response or VL over the course of the study. Although no significant sex-based difference in virologic response rates was observed in the GRACE study, black patients did have lower response rates than white or Hispanic patients [8, 12]. Based on the results of this study, however, this lower response rate is not due to differences in pharmacokinetic profiles between racial groups. Even though there was no significant relationship seen between darunavir pharmacokinetic parameters and safety, it is possible that the slightly higher ritonavir exposure in women may contribute to the small sex-based differences in adverse events reported in the GRACE study; women reported slightly higher rates of nausea and vomiting, whereas men had higher rates of diarrhea [8].

A relationship between etravirine pharmacokinetic parameters and efficacy was observed in this study. Patients with etravirine AUC_12h_ or C_0h_ in the lowest quartile (≤2712 ng*·*h/mL or ≤160 ng/mL, resp.) had the smallest change in log_10_ VL from baseline to Week 48 and the lowest response rates, compared with the other pharmacokinetic quartiles. The response rates of patients in the lowest quartile of etravirine AUC_12h_ were similar in the GRACE and DUET trials (56.7% and 59.0%, resp.; nonvirologic-censored populations; data on file). GRACE was a single-armed study, so it is difficult to determine whether having low pharmacokinetic etravirine exposure itself or a factor contributing to low pharmacokinetic etravirine exposure, such as nonadherence, is contributing to the lower response rates in this group. No relevant relationship between etravirine pharmacokinetic parameters and the safety of etravirine was seen in this study.

Pharmacokinetic results from GRACE demonstrated that darunavir and etravirine exposures are not substantially affected by sex and race, and that the darunavir C_0h_ was above the protein-binding corrected median effective concentration (EC_50_) value for PI-resistant virus for all patients. These results suggest that darunavir/ritonavir and etravirine therapy are effective in treatment-experienced men and women and across races. Furthermore, no relevant relationship was seen between darunavir pharmacokinetic parameters and a range of extrinsic and intrinsic covariates, or the efficacy or safety of darunavir/ritonavir-based therapy. The response rate obtained by those patients with the lowest etravirine exposures in this study was substantially higher than the response rate of patients in DUET who received no etravirine [24], suggesting that etravirine use may still be beneficial to treatment-experienced patients with lower etravirine exposure. It should be noted that this study was conducted using darunavir/ritonavir 600/100 mg twice daily, which was the approved dose for treatment-experienced patients at the time of the study. Since then, darunavir/ritonavir 800/100 mg once daily has been approved for treatment-experienced patients with no darunavir resistance-associated mutations, based upon results from the ODIN trial [6, 25]. Further pharmacokinetic analyses will therefore be needed for this newly approved dose.

## 5. Conclusion

These findings support the results from the overall GRACE trial, which showed that darunavir/ritonavir-based therapy is generally safe and effective and that etravirine use is associated with improved outcomes [8, 13], and suggest that darunavir/ritonavir and etravirine may be administered without dose adjustment in both sexes and across races.

##  Disclosures 

T. N. Kakuda, V. Sekar, B. Coate, and R. Ryan are employees of Janssen Research & Development, LLC. P. Vis is an employee of Janssen Infectious Diseases, BVBA. D. Anderson, G. D. L. Rosa, and J. Mrus are employees of Janssen Services, LLC.

## Figures and Tables

**Table 1:** Baseline demographics and disease characteristics for the pharmacokinetic population (overall and etravirine populations).

Parameter	Overall *N* = 376	Etravirine subgroup *n* = 190
Sex, *n* (%)		
Male	128 (34.0)	82 (43.2)
Female	248 (66.0)	108 (56.8)
Race, *n* (%)		
Black	226 (60.1)	122 (64.2)
Hispanic	84 (22.3)	33 (17.4)
White	62 (16.5)	31 (16.3)
Asian/other	4 (1.1)	4 (2.1)
Median (range) age, years	43.0 (19.0, 78.0)	45.0 (19.0, 78.0)
Mean (SE) weight, kg	76.7 (1.03)	76.8 (1.50)
Mean (SE) BMI, kg/m^2^	27.3 (0.35)^a^	27.0 (0.50)
Mean (SE) duration of HIV infection, years	11.3 (0.29)^a^	12.5 (0.38)^b^
Mean (SE) HIV-1 RNA, log_10_ copies/mL	4.64 (0.044)	4.60 (0.067)
Median (range) CD4^+^ count, cells/mm^3^	203 (2, 1125)	186 (1, 1125)
CDC Class C, *n* (%)	148 (39.4)	87 (45.8)
Median (range) darunavir, fold change^c^	0.6 (0.3, 607.9)^d^	0.6 (0.3, 607.9)
Median (range) etravirine, fold change^c^	1.3 (0.3, 93.8)^d^	1.4 (0.3, 93.8)
Prior use of ≥2 PIs, *n* (%)	228 (60.6)	135 (71.1)

^a^ *n* = 375. ^b^ *n* = 189. ^c^virco^®^TYPE HIV-1 resistance analysis; patients were considered susceptible to darunavir if the fold change was <3.4 and to etravirine if the fold change was <3.2. ^d^ *n* = 374; 2 patients, one Hispanic and one white (both women), did not have resistance testing at baseline. SE: standard error; BMI: body mass index; CDC: United States Center for Disease Control and Prevention; PI: protease inhibitor.

**Table 2:** Population pharmacokinetics at Week 48 (univariate analysis).

	*n*	Darunavir		*n*	Etravirine	
		AUC_12h_ Median (range) Ng·h/mL	C_0h_ Median (range) ng/mL		AUC_12h_ Median (range) ng·h/mL	C_0h_ Median (range) ng/mL
Overall population	376	60,642 (26,117–128,790)	3624 (931–9570)	190	4183 (212–27,960)	280 (4–2211)
Age, years						
≤30	39	58,309 (33,050–128,790)	3317 (1145–9570)	17	3476 (568–5261)	212 (5–331)
>30 to ≤50	260	59,955 (26,117–105,130)	3584 (931–6841)	128	4348 (212–27,960)	286 (4–2211)
>50 to ≤65	68	64,337 (40,299–120,880)	3957 (2215–8906)	38	4366 (295–11,684)	291 (11–890)
>65	9	63,978 (38,171–84,295)	3916 (1879–5869)	7	7484 (2213–17,921)	541 (126–1392)
Weight at baseline, kg						
≤62.33	94	61,005 (32,271–128,790)	3665 (1169–9570)	46	3675 (295–17,921)	226 (11–1392)
>62.33 to ≤73.94	96	58,367 (29,888–93,408)	3489 (931–6081)	48	3824 (1004–20,495)	250 (42–1605)
>73.94 to ≤87.09	92	63,942 (34,692–105,130)	3903 (1568–6502)	52	4960 (212–27,960)	332 (4–2211)
>87.09	94	61,090 (26,117–100,710)	3561 (1258–6943)	44	4638 (1319–18,977)	314 (60–1487)
Hepatitis B co-infection status						
No	362	60,831 (26,117–128,790)	3618 (931–9570)	184	4141 (212–27,960)	278 (4–2211)
Yes	14	57,936 (37,506–97,125)	3718 (1640–6784)	6	5504 (3751–11,684)	382 (241–890)
Use of TDF						
No	58	61,443 (38,104–109,410)	3489 (1169–7473)	45	5051 (295–17,921)	329 (11–1392)
Yes	318	60,601 (26,117–128,790)	3627 (931–9570)	145	3998 (212–27,960)	258 (4–2211)
Use of etravirine						
No	187	58,933 (26,117–128,790)	3489 (1036–9570)	NA	NA	NA
Yes	189	62,626 (30,960–109,410)	3806 (931–7473)	NA	NA	NA

AUC_12h_: area under the plasma concentration–time curve over 12 hours; C_0h_: trough concentration; TDF: tenofovir disoproxil fumarate; NA: not applicable.

**Table 3:** Relationship of selected covariates with darunavir or etravirine pharmacokinetic parameters at Week 48—analysis of covariance.

	Darunavir				Etravirine			
Covariate	Relationship to AUC_12h_, estimate (SE)	Relationship to AUC_12h_, adjusted *P* value	Relationship to C_0h_, estimate (SE)	Relationship to C_0h_, adjusted *P* value	Relationship to AUC_12h_, estimate (SE)	Relationship to AUC_12h_, adjusted *P* value	Relationship to C_0h_, estimate (SE)	Relationship to C_0h_, adjusted *P* value
Sex	0.028 (0.011)	0.011	0.050 (0.016)	0.002	−0.035 (0.049)	0.479	−0.049 (0.063)	0.432

Race^a^		0.246		0.115		0.808		0.843

Asian	–0.099 (0.096)		−0.136 (0.143)		0.369 (0.331)		0.452 (0.422)	
Black	–0.002 (0.068)		0.017 (0.101)		0.116 (0.236)		0.155 (0.301)	
Hispanic	0.004 (0.069)		0.041 (0.103)		0.148 (0.242)		0.183 (0.308)	
White	0.023 (0.069)		0.058 (0.103)		0.130 (0.240)		0.184 (0.306)	
Other	0.000		0.000		0.000		0.000	

Age,^b^ years	0.001 (0.001)	0.005	0.003 (0.001)	<0.001	0.005 (0.002)	0.029	0.007 (0.003)	0.023

Weight,^b^ kg	0.000 (0.000)	0.839	0.000 (0.000)	0.784	0.002 (0.001)	0.179	0.002 (0.002)	0.149

Use of TDF	–0.019 (0.014)	0.168	–0.038 (0.021)	0.072	NE	NE	NE	NE

Use of etravirine	–0.024 (0.028)	0.389	–0.032 (0.042)	0.450	NA	NA	NA	NA

^a^Five-way comparison: white, black, Hispanic, Asian, and other. ^b^Modeled as continuous linear variables.   AUC_12h_: area under the plasma concentration–time curve over 12 hours; SE: standard error; C_0h_: trough concentration; TDF: tenofovir disoproxil fumarate; NE: not evaluated; NA: not applicable.

**Table 4:** Pharmacokinetics of darunavir, ritonavir, and etravirine (intense pharmacokinetic sampling).

	Week 4				Week 24				Week 48			
Mean ± SD	AUC_12h_, ng·h/mL	*n*	C_0h_, ng/mL	*n*	AUC_12h_, ng·h/mL	*n*	C_0h_, ng/mL	*n*	AUC_12h_, ng·h/mL	*n*	C_0h_, ng/mL	*n*
						Darunavir						

Overall	62,360 ± 25,020	32	3559 ± 2385	26	62,230 ± 27,420	22	5042 ± 2080	20	56,320 ± 22,440	21	3388 ± 2078	21
Men												
Overall	55,570 ± 21,220	10	3863 ± 2402	8	51,510 ± 30,260	8	4765 ± 2240	6	52,040 ± 25,010	9	3406 ± 2134	9
Black	59,410 ± 26,060	6	4445 ± 3006	4	57,690 ± 36,900	5	5175 ± 2771	4	61,300 ± 28,130	4	3836 ± 2268	5
Hispanic	48,110 ± 14,160	3	3280 ± 1875	4	41,210 ± 15,070	3	3945 ± 134	2	35,630 ± 11,440	4	1973 ± 1444	3
White	54, 910	1	—	—	—	—	—	—	80, 630	1	5550	1
Women												
Overall	65,440 ± 26,450	22	3424 ± 2434	18	68,360 ± 24,710	14	5160 ± 2085	14	59,540 ± 20,850	12	3375 ± 2131	12
Black	66,290 ± 29,930	16	3050 ± 2540	14	63,500 ± 22,080	11	5723 ± 1876	10	61,950 ± 23,570	9	3418 ± 2127	8
Hispanic	61,980 ± 17,070	5	4877 ± 1968	3	86,170 ± 30,410	3	3753 ± 2139	4	52,290 ± 7973	3	3290 ± 2465	4
White	69,090	1	4310	1	—	—	—	—	—	—	—	—

						Ritonavir						

Overall	5722 ± 3788	32	235 ± 223	26	6473 ± 4561	22	402 ± 326	20	5340 ± 2788	21	281 ± 187	21
Men												
Overall	4399 ± 1876	10	276 ± 227	8	5047 ± 3409	8	296 ± 158	6	5611 ± 3480	9	318 ± 198	9
Black	5060 ± 1676	6	342 ± 260	4	6216 ± 3917	5	343 ± 143	4	7633 ± 3297	4	358 ± 171	5
Hispanic	3625 ± 2290	3	210 ± 204	4	3099 ± 941	3	201 ± 192	2	2833 ± 1620	4	180 ± 202	3
White	2755	1	—	—	—	—	—	—	8639	1	537	1
Women												
Overall	6324 ± 4297	22	217 ± 225	18	7287 ± 5038	14	448 ± 371	14	5136 ± 2286	12	252 ± 182	12
Black	5951 ± 4701	16	146 ± 149	14	7091 ± 5577	11	471 ± 436	10	4773 ± 2354	9	209 ± 136	8
Hispanic	6825 ± 3191	5	457 ± 354	3	8007 ± 2919	3	390 ± 144	4	6226 ± 2048	3	339 ± 251	4
White	9782	1	491	1	—	—	—	—	—	—	—	—

						Etravirine						

Overall	6980 ± 4205	16	455 ± 238	14	5495 ± 3232	10	460 ± 319	13	5520 ± 2756	9	375 ± 215	12
Men												
Overall	6636 ± 5720	8	409 ± 305	6	4020 ± 1673	4	410 ± 351	6	5694 ± 3729	5	382 ± 278	6
Black	5919 ± 5840	4	563 ± 392	3	2805 ± 888	2	265 ± 209	2	2027	1	312 ± 338	2
Hispanic	4165 ± 583	3	255 ± 88	3	5235 ± 1306	2	276 ± 14	3	4819 ± 383	3	273 ± 73	3
White	16,910	1	—	—	—	—	1100	1	11, 990	1	846	1
Women												
Overall	7323 ± 2212	8	490 ± 188	8	6479 ± 3771	6	503 ± 311	7	5304 ± 1265	4	369 ± 156	6
Black	7381 ± 2383	7	465 ± 187	7	5902 ± 3910	5	456 ± 313	6	5304 ± 1265	4	392 ± 162	5
Hispanic	6918	1	670	1	9362	1	783	1	—	—	253	1

SD: standard deviation; AUC_12h_: area under the plasma concentration–time curve over 12 hours; C_0h_: trough concentration.
